# Limitations of Correlation-Based Inference in Complex Virus-Microbe Communities

**DOI:** 10.1128/mSystems.00084-18

**Published:** 2018-08-28

**Authors:** Ashley R. Coenen, Joshua S. Weitz

**Affiliations:** a School of Biological Sciences, Georgia Institute of Technology, Atlanta, Georgia, USA; b School of Physics, Georgia Institute of Technology, Atlanta, Georgia, USA; Vanderbilt University

**Keywords:** correlation, inference, interaction network, microbial ecology, viral ecology

## Abstract

Microbes are present in high abundances in the environment and in human-associated microbiomes, often exceeding 1 million per ml. Viruses of microbes are present in even higher abundances and are important in shaping microbial populations, communities, and ecosystems. Given the relative specificity of viral infection, it is essential to identify the functional linkages between viruses and their microbial hosts, particularly given dynamic changes in virus and host abundances. Multiple approaches have been proposed to infer infection networks from time series of *in situ* communities, among which correlation-based approaches have emerged as the *de facto* standard. In this work, we evaluate the accuracy of correlation-based inference methods using an *in silico* approach. In doing so, we compare predicted networks to actual networks to assess the self-consistency of correlation-based inference. At odds with assumptions underlying its widespread use, we find that correlation is a poor predictor of interactions in the context of viral infection and lysis of microbial hosts. The failure to predict interactions holds for methods that leverage product-moment, time-lagged, and relative-abundance-based correlations. In closing, we discuss alternative inference methods, particularly model-based methods, as a means to infer interactions in complex microbial communities with viruses.

**IMPORTANCE** Inferring interactions from population time series is an active and ongoing area of research. It is relevant across many biological systems—particularly in virus-microbe communities, but also in gene regulatory networks, neural networks, and ecological communities broadly. Correlation-based inference—using correlations to predict interactions—is widespread. However, it is well-known that “correlation does not imply causation.” Despite this, many studies apply correlation-based inference methods to experimental time series without first assessing the potential scope for accurate inference. Here, we find that several correlation-based inference methods fail to recover interactions within *in silico* virus-microbe communities, raising questions on their relevance when applied *in situ*.

## Abstract (precis)

Inferring interactions from population time series is an active and ongoing area of research. It is relevant across many biological systems—particularly in virus-microbe communities, but also in gene regulatory networks, neural networks, and ecological communities broadly. Correlation-based inference—using correlations to predict interactions—is widespread. However, it is well-known that “correlation does not imply causation.” Despite this, many studies apply correlation-based inference methods to experimental time series without first assessing the potential scope for accurate inference. Here, we find that several correlation-based inference methods fail to recover interactions within *in silico* virus-microbe communities, raising questions on their relevance when applied *in situ*.

## INTRODUCTION

Viruses of microbes are ubiquitous and highly diverse in marine, soil, and human-associated environments. Viruses interact with their microbial hosts in many ways. For example, they can transfer genes between microbial hosts (1, 2), alter host physiology and metabolism (3, 4), and redirect the flow of organic matter in food webs through cell lysis (5, 6). Viruses are important parts of microbial communities, and characterizing the interactions between viruses and their microbial hosts is critical for understanding microbial community structure and ecosystem function (5, 7–9).

A key step in characterizing virus-microbe interactions is determining which viruses can infect which microbes. Viruses are known to be relatively specific but not exclusive in their microbial host range. Individual viruses may infect multiple strains of an isolated microbe, or they may infect across genera as part of complex virus-microbe interaction networks (10, 11). For example, cyanophage can infect both *Prochlorococcus* and *Synechococcus*, which are two distinct genera of marine cyanobacteria (12). However, knowledge of viral host range remains limited, because existing experimental methods for directly testing for viral infection are generally not applicable to an entire *in situ* community. Culture-based methods such as plaque assays are useful for checking for viral infection at the strain level and permit high confidence in their results, but they are not broadly applicable, as many viruses and microbes are difficult or currently impossible to isolate and culture (1). Partially culture-independent methods, such as viral tagging (13, 14) and digital PCR (15), overcome some of these hurdles but only for particular targetable viruses and microbes. Similarly, single-cell genome analysis is able to link individual viruses to microbial hosts (16–18) but for a relatively small number of cells.

Viral metagenomics offers an alternate route for probing virus-microbe interactions for entire *in situ* communities, bypassing culturing altogether (19–21). The viral sequences obtained from metagenomes can be analyzed directly using bioinformatics-based methods to predict microbial hosts (22, 23), although such methods may be appropriate only for a subset of viruses (phages and archaeal viruses but not eukaryotic viruses) and putative hosts (prokaryotes but not eukaryotes). Alternatively, metagenomic sampling of a community over time can provide estimates of the changing abundances of viral and microbial populations at high resolution in time and across taxonomic groups. Once these high-resolution time series are obtained, they can be used to predict virus-microbe interactions using a variety of statistical and mathematical inference methods (for reviews, see references 24 to 28).

Correlation and correlation-based methods are among the most widely used network inference methods for microbial communities (25). For example, extended local similarity analysis (eLSA) is a correlation-based method that allows for both local and time-lagged correlations (29–31), and it has been used to infer interaction networks in communities of marine bacteria (32, 33), bacteria and phytoplankton (34, 35), bacteria and viruses (36), and bacteria, viruses, and protists (37, 38). In addition, several correlation-based methods have been developed to address challenges associated with the compositional nature of “-omics” data sets (25, 39), including sparse correlations for compositional data (SparCC) (40).

Regardless of the particular details of these methods, all correlation-based inference operates on the same core assumptions that interacting populations trend together (are correlated) and that noninteracting populations do not trend together (are not correlated). Particular correlation-based methods may relax or augment this assumption. For example, with eLSA, the trends may be time lagged (29–31); with simple rank correlations, the trends may be nonparametric; and with compositional methods like SparCC, the trends may occur between ratios of relative abundances (40). In communities with only a few populations and simple interactions, population trends may indeed be indicative of ecological mechanism. In these contexts, some correlation-based methods have been shown to recapitulate microbe-microbe interactions with limited success (25). Typically, however, the challenge of inferring interaction networks applies to diverse communities and complex ecological interactions. Microbial communities often have dozens, hundreds, or more distinct populations, each of which may interact with many other populations through nonlinear mechanisms such as viral lysis, as well as be influenced by fluctuating abiotic drivers. In these contexts, the relationship between correlation and ecological mechanism is poorly understood. Often, correlations do not have a simple mechanistic interpretation, a well-known adage (“correlation does not imply causation”) that is often disregarded.

Despite the challenge of interpretation, correlation-based inference methods are widely used with *in situ* data sets (25, 29–40). Benchmarking inferred networks—connecting correlations to specific ecological mechanisms—is difficult. In the context of lytic infections of environmental microbes by viruses, there is (usually) no existing “gold standard” interaction network with which to validate inferred interactions. Therefore, in this work, we take an *in silico* approach to assess the accuracy of correlation-based inference. To do this, we simulate virus-microbe community dynamics with an interaction network which is prescribed *a priori* and use it to benchmark inferred networks. Several existing studies have applied similar *in silico* approaches in the case of both microbe-microbe and microbe-virus interactions and found that simple Pearson correlation (39, 41) and several correlation-based methods (25) either fail or are inconsistent in recapitulating interaction networks. Here, we provide an in-depth assessment of the potential for correlation-based inference in diverse communities of microbes and viruses. As we show, correlation-based inference fails to recapitulate virus-microbe interactions and performs worse in more diverse communities. The failure of correlation-based inference in this context raises concerns over its use in inferring microbe-parasite interactions as well as microbe-predator and microbe-microbe interactions more broadly.

## RESULTS

### Standard Pearson correlation.

We calculated the standard Pearson correlation networks for an ensemble of *in silico* communities that varied in network size and network structure. For each network size *N* = 10, 25, 50, we generated 20 unique interaction networks. Ten of the networks were generated so that they were distributed along a range of nestedness values, and the other ten were generated so that they were distributed along a range of modularity values (see “Generating interaction networks and characterizing network structure” in Materials and Methods). For each interaction network, a single set of life history traits were generated to ensure coexistence using biologically feasible ranges (see “Choosing life history traits for coexistence” in Materials and Methods). The mechanistic model for the community dynamics is described below in “Dynamic model of a virus-microbe community.” Time series were simulated according to “Simulating and sampling time series” with δ = 0.3, that is, the initial conditions were the fixed-point values perturbed by 30% (for additional values of δ, see Fig. S4 in the supplemental material). For δ = 0.3, the mean coefficient of variation was 12% for host time series and 4% for virus time series (Fig. S1). The time series were sampled during the transient dynamics to represent *in situ* communities which are likely perturbed from equilibrium due to changing environmental conditions and intrinsic feedback. We sampled the time series every 2 h for 200 h, that is, we took 100 samples (for additional sample frequencies, see Fig. S7).

10.1128/mSystems.00084-18.1 FIG S1  Distributions of coefficients of variation for each simulated host time series (top row) and virus time series (bottom row) for the ensemble of communities over three network sizes (*N* = 10, 25, 50 with 20 communities for each *N*). The coefficient of variation (CV) for an individual time series is CV = σ/μ where σ is the standard deviation and μ is the mean of the time series from *t* = 0 h to *t* = 200 h (the sample duration used in the main text). The colors correspond to time series with different initial condition perturbation amounts (δ = 0.1 [blue], 0.3 [orange], 0.5 [yellow]); the three distributions are plotted cumulatively here. Solid vertical lines correspond to distribution means. For both hosts and viruses, CV scales with δ but does not scale with *N*. The mean CVs for host time series for δ = 0.1, 0.3, 0.5 (averaged across network sizes) are 0.04 (10^−1.40^), 0.12 (10^−0.92^), and 0.22 (10^−0.67^), respectively. For virus time series, they are 0.01 (10^−1.88^), 0.04 (10^−1.41^), and 0.06 (10^−1.20^). Notably, increasing δ (and thus CV) did not improve AUC for any of the correlation-based inference methods (Fig. S4, S5, and S6). Download FIG S1, TIF file, 0.2 MB. Copyright © 2018 Coenen and Weitz. 2018 Coenen and Weitz This content is distributed under the terms of the Creative Commons Attribution 4.0 International license.

**FIG 1:**
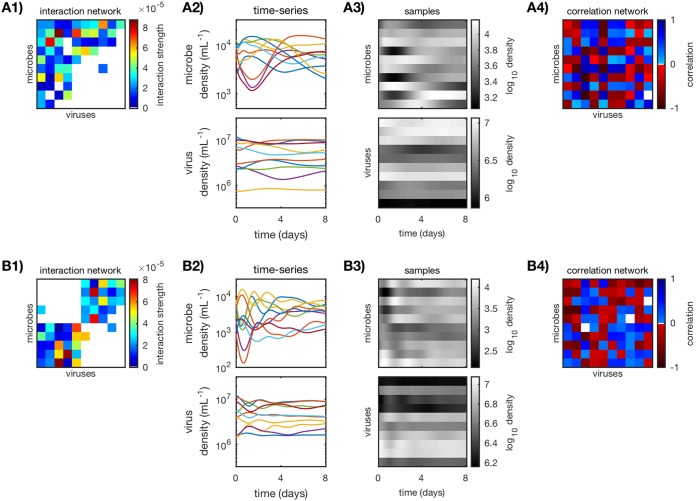
Calculating standard Pearson correlation networks for an *in silico* nested (A) and a modular (B) community (*N* = 10). (A1 and B1) Original weighted interaction networks, generated as described in “Generating interaction networks and characterizing network structure” and “Choosing life history traits for coexistence” in Materials and Methods. (A2 and B2) Simulated time series of the virus-microbe dynamic system as described in “Simulating and sampling time series” (δ = 0.3). (A3 and B3) Log-transformed samples, sampled every 2 h for 200 h from the simulated time series. (A4 and B4) Pearson correlation networks, calculated from log-transformed samples as described in “Standard and time-delayed Pearson correlation networks.”

**FIG 2:**
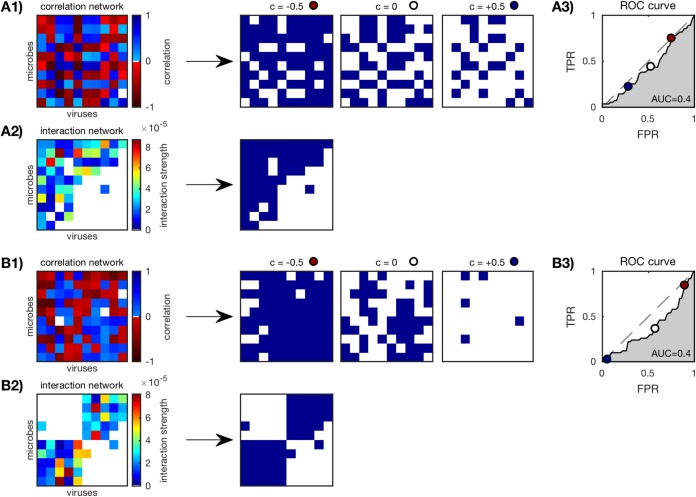
Scoring correlation network accuracy of an *in silico* nested (A) and a modular (B) community (*N* = 10; see Fig. 1) as described in “Scoring correlation network accuracy” in Materials and Methods. (A1 and B1) Correlation networks are binarized according to thresholds *c* between −1 and +1, three of which are shown here (*c* = −0.5, 0, and 0.5). (A2 and B2) Original interaction networks are also binarized. (A3 and B3) True-positive rate (TPR) versus false-positive rate (FPR) of the binarized correlation networks for each threshold *c*. Three example thresholds (*c* = −0.5, 0, and 0.5) are marked (red, white, and dark blue circles). The “nondiscrimination” line (gray dashed line) is where TPR = FPR. The AUC or area under the ROC is a measure of relative TPR to FPR over all thresholds; AUC = 1 is a perfect result.

For each *in silico* community, we calculated the standard Pearson correlation network as described in “Standard and time-delayed Pearson correlation networks” in Materials and Methods. Two examples of *in silico* communities of size *N* = 10 are shown in Fig. 1 with their simulated time series, log-transformed samples, and resulting correlation networks. The correlation networks were scored against the original interaction networks by computing area under the curve (AUC) as described in “Scoring correlation network accuracy”. The procedure for computing AUC is shown in Fig. 2 for the two examples of *in silico* communities.

**FIG 3:**
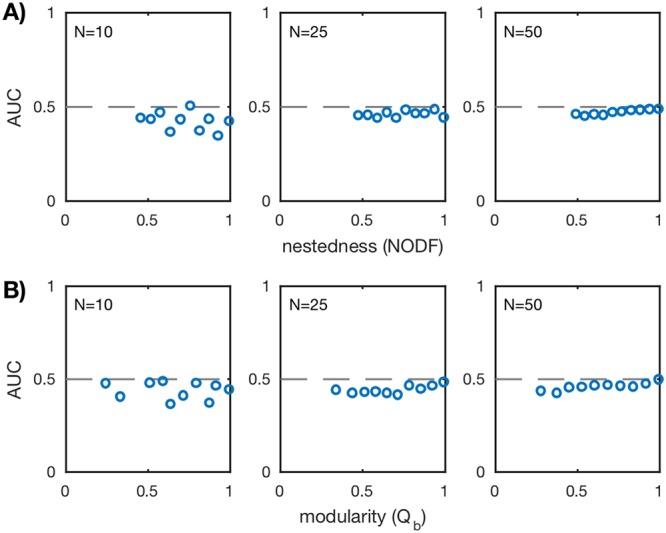
AUC values for standard Pearson correlation for the ensemble of nested (A) and modular (B) communities over three network sizes *N* = 10, 25, 50 (20 communities for each network size). AUC is computed as described in “Scoring correlation network accuracy” in Materials and Methods. Each plotted point corresponds to a unique *in silico* community. The dashed lines mark AUC = 1/2 and imply that the predicted network did no better than random guessing.

AUC values for all *in silico* communities are shown in Fig. 3. Across different network sizes and network structures, the AUC is approximately 1/2, implying that standard Pearson correlation networks lack predictive power. Similar results were found when changing the initial condition perturbation δ (Fig. S4) and the sampling frequency (Fig. S7). There are some instances where the AUC does deviate from 1/2 for the smaller networks (*N* = 10), although these deviations are small (≈±10%). Interestingly, these deviations tend to be negative, indicating a misclassification of the interaction condition, that is, negative correlations are slightly better predictors of interaction than positive correlations. Overall, however, the deviations disappear for larger networks (*N* = 50), implying that they are exceptions rather than the norm. We completed identical analyses for additional correlation metrics, in particular Spearman correlation and Kendall correlation (see Fig. S2). We found similar results, reinforcing our conclusion that simple correlations between time series are poor predictors of the underlying interaction network.

10.1128/mSystems.00084-18.2 FIG S2  AUC values for standard correlation of various types (Pearson correlation [blue], Spearman correlation [orange], and Kendall correlation [yellow]) for the ensemble of nested (A) and modular (B) communities over three network sizes *N* = 10, 25, 50. The dashed lines mark AUC = 1/2 and imply that the predicted network did no better than random guessing. This figure corresponds to Fig. 3 in the main manuscript. Download FIG S2, TIF file, 0.1 MB. Copyright © 2018 Coenen and Weitz. 2018 Coenen and Weitz This content is distributed under the terms of the Creative Commons Attribution 4.0 International license.

### Time-delayed Pearson correlation.

Given the results of the previous section (“Standard Pearson correlation”)—that standard correlations do not recapitulate interactions—we computed time-delayed correlation networks for the same ensemble of *in silico* communities. The addition of time delays to standard correlation approaches is motivated by a large body of theoretical work on predator-prey dynamics, where both predator and prey populations oscillate but with a phase delay between them (42). Similar results hold for the phase delay in simple phage-bacteria dynamics (43). Time-delayed correlations are the basis of several existing correlation-based inference methods, including eLSA (29–31).

**FIG 4:**
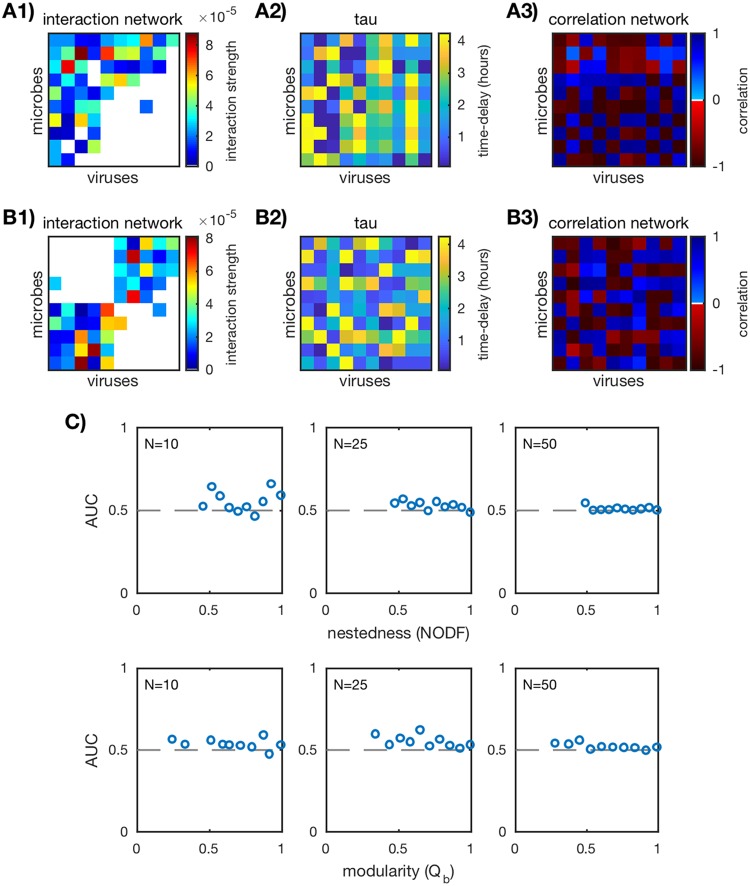
Performance of time-delayed Pearson correlation. (A1 and B1) Two examples of *in silico* interaction networks (*N* = 10). (A2 and B2) Time delays τ_*ij*_ for each virus-host pair, chosen so that the absolute value of the correlation is maximized. (A3 and B3) Time-delayed Pearson correlation networks calculated as described in “Standard and time-delayed Pearson correlation networks” in Materials and Methods. (C) AUC values for the ensemble of nested (top row) and modular (bottom row) communities over three network sizes *N* = 10, 25, 50 (20 communities for each network size). Each plotted point corresponds to a unique *in silico* community. The dashed lines mark AUC = 1/2 and imply that the predicted network did no better than random guessing.

For this analysis, we used the same ensemble of *in silico* communities (networks with network sizes *N* = 10, 25, 50 and different levels of nestedness and modularity), simulated time series (δ = 0.3; see Fig. S5 in the supplemental material), and sample frequency (2 h; Fig. S8) as before (see “Standard Pearson correlation” above for time series). We calculated the time-delayed Pearson correlation networks as described in “Standard and time-delayed Pearson correlation networks” below, where for each virus-host pair, virus *j* is sampled later in time relative to host *i* by the time delay value τ_*ij*_ (for Spearman correlation and Kendall correlation, see Fig. S3). Each delay is chosen such that the absolute value of the correlation for the virus-host pair is maximized. Since the optimal time delay is not known in advance, delays between 0 h and half the sample length *t_s_* (*t_s_*/2 = 100 h) were considered. The number of samples used to compute each correlation coefficient was kept fixed at *S* = 100 (sample duration, 200 h). Time-delayed Pearson correlation networks for the two example *in silico* communities of size *N* = 10 are shown in Fig. 4A and B. AUC was computed as described in “Scoring correlation network accuracy” below.

10.1128/mSystems.00084-18.3 FIG S3  AUC values for time-delayed correlation of various types (Pearson correlation [blue], Spearman correlation [orange], and Kendall correlation [yellow]) for the ensemble of nested (A) and modular (B) communities over three network sizes *N* = 10, 25, 50. The dashed lines mark AUC = 1/2 and imply that the predicted network did no better than random guessing. This figure corresponds to Fig. 4 in the main manuscript. Download FIG S3, TIF file, 0.1 MB. Copyright © 2018 Coenen and Weitz. 2018 Coenen and Weitz This content is distributed under the terms of the Creative Commons Attribution 4.0 International license.

10.1128/mSystems.00084-18.4 FIG S4  AUC values for standard Pearson correlation with different δ values (δ = 0.1 [blue], 0.3 [orange], and 0.5 [yellow]) for the ensemble of nested (A) and modular (B) communities over three network sizes *N* = 10, 25, 50. The dashed lines mark AUC = 1/2 and imply that the predicted network did no better than random guessing. This figure corresponds to Fig. 3 in the main manuscript. Download FIG S4, TIF file, 0.1 MB. Copyright © 2018 Coenen and Weitz. 2018 Coenen and Weitz This content is distributed under the terms of the Creative Commons Attribution 4.0 International license.

10.1128/mSystems.00084-18.5 FIG S5  AUC values for time-delayed Pearson correlation with different δ values (δ = 0.1 [blue], 0.3 [orange], and 0.5 [yellow]) for the ensemble of nested (A) and modular communities (B) over three network sizes *N* = 10, 25, 50. The dashed lines mark AUC = 1/2 and imply that the predicted network did no better than random guessing. This figure corresponds to Fig. 4 in the main manuscript. Download FIG S5, TIF file, 0.1 MB. Copyright © 2018 Coenen and Weitz. 2018 Coenen and Weitz This content is distributed under the terms of the Creative Commons Attribution 4.0 International license.

**FIG 5:**
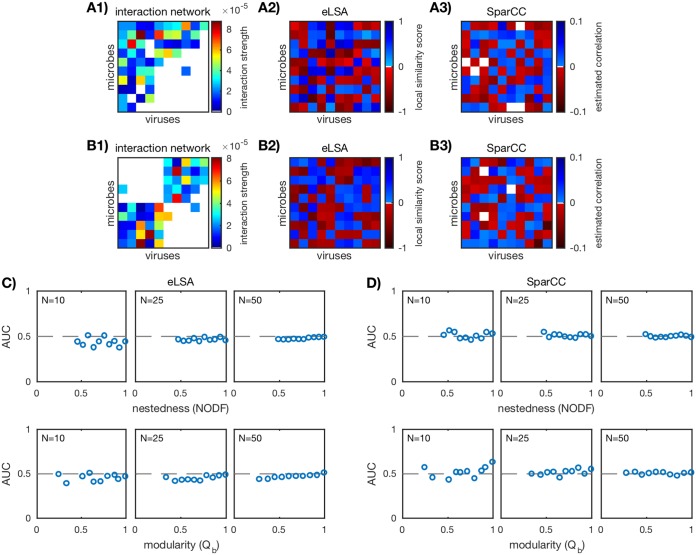
Performance of correlation-based inference methods eLSA and SparCC. (A1 and B1) Two examples of *in silico* interaction networks (*N* = 10). (A2 and B2) eLSA-predicted network computed as described in “eLSA networks” in Materials and Methods. (A3 and B3) SparCC-predicted network computed as described in “SparCC networks” (color bar adjusted for visibility). (C and D) AUC values for the ensemble of nested (top row) and modular (bottom row) communities over three network sizes *N* = 10, 25, 50 (20 communities for each network size). Each plotted point corresponds to a unique *in silico* community. The dashed lines mark AUC = 1/2 and imply that the predicted network did no better than random guessing.

10.1128/mSystems.00084-18.6 FIG S6  AUC values for eLSA and SparCC with different δ values (δ = 0.1 [blue], 0.3 [orange], and 0.5 [yellow]) for the ensemble of nested (A) and modular communities (B) over three network sizes *N* = 10, 25, 50. The dashed lines mark AUC = 1/2 and imply that the predicted network did no better than random guessing. This figure corresponds to Fig. 5 in the main manuscript. Download FIG S6, TIF file, 0.5 MB. Copyright © 2018 Coenen and Weitz. 2018 Coenen and Weitz This content is distributed under the terms of the Creative Commons Attribution 4.0 International license.

10.1128/mSystems.00084-18.7 FIG S7  AUC values for standard Pearson correlation with different sample frequencies (0.5 h [blue], 2 h [orange], and 4 h [yellow]) for the ensemble of nested (A) and modular communities (B) over three network sizes *N* = 10, 25, 50. The dashed lines mark AUC = 1/2 and imply that the predicted network did no better than random guessing. This figure corresponds to Fig. 3 in the main manuscript. Download FIG S7, TIF file, 0.6 MB. Copyright © 2018 Coenen and Weitz. 2018 Coenen and Weitz This content is distributed under the terms of the Creative Commons Attribution 4.0 International license.

AUC values for all *in silico* communities are shown in Fig. 4C. For the small networks (*N* = 10), there are a few particular networks that have AUC scores greater than 1/2. For the remaining small networks and the large networks (*N* = 25, 50), AUC is ≈1/2, implying that time-delayed Pearson correlation lacks predictive power for these networks. Similar results were found for alternate correlation metrics (Spearman and Kendall correlations; Fig. S3), initial condition perturbations δ (Fig. S5), and sampling frequencies (Fig. S8). Because AUC deviates from 1/2 for only a few small networks and this deviation disappears for large networks, it should be considered an exception rather than the norm for time-delayed Pearson correlation.

10.1128/mSystems.00084-18.8 FIG S8  AUC values for time-delayed Pearson correlation with different sample frequencies (0.5 h [blue], 2 h [orange], and 4 h [yellow]) for the ensemble of nested (A) and modular (B) communities over three network sizes *N* = 10, 25, 50. The dashed lines mark AUC = 1/2 and imply that the predicted network did no better than random guessing. This figure corresponds to Fig. 4 in the main manuscript. Download FIG S8, TIF file, 0.9 MB. Copyright © 2018 Coenen and Weitz. 2018 Coenen and Weitz This content is distributed under the terms of the Creative Commons Attribution 4.0 International license.

### Correlation-based methods eLSA and SparCC.

We performed a similar *in silico* analysis using eLSA (29–31) and SparCC (40), two established correlation-based inference methods that are widely used with *in situ* time series data. We used the same ensemble of *in silico* communities as before (network sizes *N* = 10, 25, 50 and networks with different levels of nestedness and modularity), along with the simulated time series (δ = 0.3; see Fig. S6), sample frequency (2 h; see Fig. S9) and sample duration (200 h). We implemented eLSA and SparCC as described in “eLSA networks” and “SparCC networks,” respectively, in Materials and Methods. eLSA and SparCC predicted networks for the two examples of *in silico* communities of size *N* = 10 are shown in Fig. 5A and B. AUC was computed as before and as described in “Scoring correlation network accuracy” below.

10.1128/mSystems.00084-18.9 FIG S9  AUC values for eLSA and SparCC with different sample frequencies (0.5 h [blue], 2 h [orange], and 4 h [yellow]) for the ensemble of nested (A) and modular (B) communities over three network sizes *N* = 10, 25, 50. The dashed lines mark AUC = 1/2 and imply that the predicted network did no better than random guessing. This figure corresponds to Fig. 5 in the main manuscript. Download FIG S9, TIF file, 0.1 MB. Copyright © 2018 Coenen and Weitz. 2018 Coenen and Weitz This content is distributed under the terms of the Creative Commons Attribution 4.0 International license.

AUC values for all *in silico* communities are shown in Fig. 5C. We see the same trends as with standard correlation and time-delayed correlation (see Fig. 3 and 4). Similar results hold for different values of the initial condition perturbation δ (Fig. S6) and sampling frequency (Fig. S9). For small networks (*N* = 10), there are a few AUC scores that deviate weakly from 1/2 (≈±10%). Interestingly, AUC scores for eLSA tend to be negative, implying a misclassification of interaction. AUC converges to 1/2 as network size increases (*N* = 25, 50), indicating that the AUC scores for small networks may themselves be spurious.

## DISCUSSION

Using *in silico* virus-microbe community dynamics, we calculated correlation networks among viral and microbial population time series samples. We tested the accuracy of several different types of correlation and time-delayed correlation (Pearson, Spearman, and Kendall correlation) and existing correlation-based inference methods (eLSA and SparCC). The correlation networks for all of these implementations failed to effectively predict the original interaction networks, as quantified by the AUC score. Failure persisted across variation in network structure, network size, degree of initial condition perturbation (i.e., scaling the variability of dynamics), and sampling frequency. We therefore conclude that these correlation-based inference methods do not meaningfully predict interactions given this mechanistic model of virus-microbe community dynamics.

Earlier, we stated the core assumption of correlation-based inference—that interacting populations are correlated and that noninteracting populations are not correlated. While this core assumption may sometimes hold in small microbe-only communities with simple interaction mechanisms (25), we find that it does not necessarily hold in more-complex virus-microbe communities. Each inference method also faces challenges unique to its formulation: eLSA in particular uses a nonstationary data transformation which may induce additional spurious correlations. We considered communities with microbes and viruses that interacted through a nonlinear mechanism (infection and lysis) across a spectrum of network sizes and network structures. We found that correlation-based inference performed poorly given variation in these network properties but that there was greater variation in performance for small networks. Because this variation is relatively small and disappears for larger networks, successful predictions for small networks may themselves be spurious. Namely, for a small network (e.g., *N* < 10), there is a greater probability of randomly guessing the interactions correctly because the space of possible networks is smaller.

Our results raise concerns about the use of correlation-based methods on *in situ* data sets, since a typical community under consideration will have dozens or more interacting strains and therefore will not be in the low-diversity microbe-only regime explored by Weiss et al. (25). Additional challenges such as external environmental drivers, measurement noise, and system stochasticity must also be carefully considered before applying correlation-based methods to *in situ* data sets. Although the degree of variability of dynamics had no effect on inference quality here, it may also be an important consideration for both experimental design and choice of inference method. For example, the model-based inference method examined by Jover et al. (44) performs better when dynamics are highly variable. On the other hand, cooccurrence-based inference methods, which require samples across space instead of time, may enable inference across different baseline environmental conditions even if the dynamics within a given environment are relatively stable.

In light of the poor performance of correlation-based methods, we advocate for increased studies of model-based inference. Model-based inference methods operate by first assuming an underlying dynamic model for the community (such as the one used in this article [see equations 1 and 2 below]). The dynamic model is then used to formulate an objective function for an optimization or regression problem, where the solution is the interaction network which best describes the sampled community time series (for example, see references 39, 41, 49, 50, 51, and 52). Unlike correlation-based methods which assume that similar trends in population indicate interaction, model-based inference has the potential to be tailored to complex communities and environments while leveraging existing knowledge about ecological mechanisms. Given favorable results of *in silico* benchmarking of model-based inference methods (39, 41, 44–47), it will be important to investigate the efficacy of model-based inference methods for complex microbial and viral communities in practice.

## MATERIALS AND METHODS

### Dynamic model of a virus-microbe community.

We model the ecological dynamics of a virus-microbe community with a system of nonlinear differential equations:

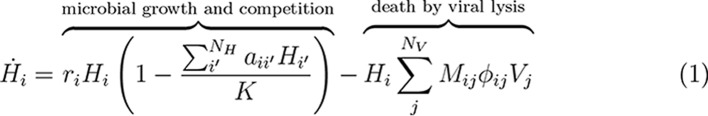


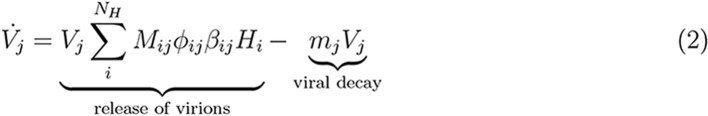

where *H_i_* and *V_j_* refer to the population density of microbial host *i* and virus *j*, respectively. There are *N_H_* different microbial host populations and *N_V_* different virus populations. For our purposes, a “population” is a group of microbes or viruses with identical life history traits, that is microbes or viruses that occupy the same functional niche.

In the absence of viruses, the microbial hosts undergo logistic growth with growth rates *r*_*i*_. The microbial hosts have a community-wide carrying capacity *K*, and they compete with each other for resources both inter- and intraspecifically with competition strength *a*_*ii*′_. Each microbial host can be infected and lysed by a subset of viruses determined by the interaction term *M*_*ij*_. If microbial host *i* can be infected by virus *j*, *M*_*ij*_ = 1; otherwise, *M*_*ij*_ = 0. The collection of all the interaction terms is the interaction network represented by matrix **M** of size *N*_*H*_ by *N*_*V*_. The adsorption rate ϕ_*ij*_ denotes how frequently microbial host *i* is infected by virus *j*.

Each virus *j*′s population grows from infecting and lysing their hosts. The rate of virus *j*′s growth is determined by its host-specific adsorption rate ϕ_*ij*_ and host-specific burst size β_*ij*_, which is the net number of new virions per infected host cell. The quantity $\overset{˜}{M}{\;}_{ij}=M_{ij}\phi_{ij}\beta_{ij}$ is the effective interaction strength between virus *j* and host *i*, and the collection of all the interaction strengths is the weighted interaction network **M̃** Finally, the viruses decay at rates *m*_*j*_.

### Generating interaction networks and characterizing network structure.

Virus-microbe interaction networks, denoted **M**, are represented as bipartite networks or matrices of size *N*_*H*_ by *N*_*V*_ where *N*_*H*_ is the number of microbial host populations and *N*_*V*_ is the number of virus populations. The element *M*_*ij*_ is 1 if microbe population *i* and virus population *j* interact and 0 if the two populations do not interact. In this paper, we consider only square networks (*N* = *N*_*H*_ = *N*_*V*_), although the analysis is easily extended to rectangular networks. We consider three network sizes *N* = 10, 25, 50.

**FIG 6:**
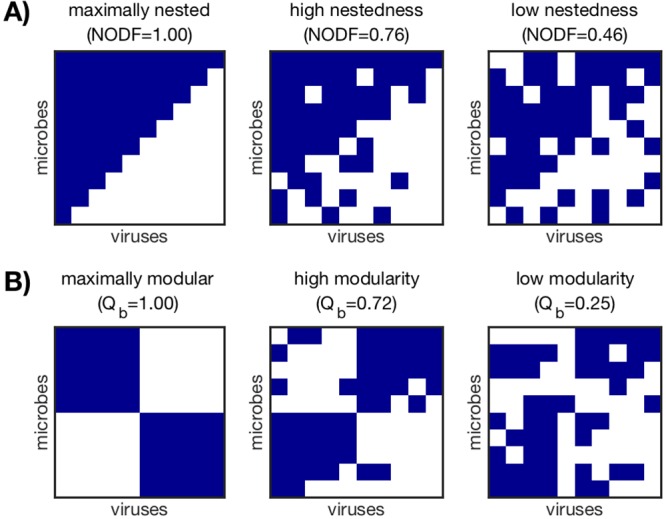
Examples of interaction networks characterized by nestedness (A) and modularity (B). The networks shown here have size *N* = 10 and fill *F* = 0.55 (A) and *F* = 0.5 (B). Within each network, rows represent microbe populations and columns represent virus populations, while navy squares indicate interaction (*M*_*ij*_ = 1). Networks were generated as described in “Generating interaction networks and characterizing network structure” in Materials and Methods. Nestedness (NODF) and modularity (*Q*_*b*_) were measured with the BiMat package and are arranged in their most nested or most modular forms (48).

For each network size *N*, we generate an ensemble of networks with different degrees of nestedness and modularity (Fig. 6). We first generate the maximally nested (Fig. 6A) and maximally modular (Fig. 6B) networks of size *N* using the BiMat Matlab package (48). In order to achieve maximal nestedness and modularity, the network fill *F* (fraction of interacting pairs) is fixed at *F* = 0.55 for the nested networks and *F* = 0.5 for the modular networks. For the modular networks, the number of modules is set to 2, 5, and 10 for the three network sizes, respectively.

To generate networks that vary in nestedness and modularity, we perform the following “rewiring” procedure. Beginning with the maximally nested or maximally modular network, we randomly select an interacting virus-microbe pair (*M*_*ij*_ = 1) and a noninteracting virus-microbe pair (*M*_*i*′*j*′_ = 0) and exchange their values. We do not allow exchanges that would result in an all-zero row or column, as that would isolate the microbe or virus population from the rest of the community. We continue the random selection of pairs without replacement until the desired nestedness or modularity has been achieved. To calculate nestedness and modularity, we use the default algorithms in the BiMat Matlab package. The nestedness metric used is NODF (nestedness metric based on overlap and decreasing fill) (49), and the algorithm used to calculate modularity is AdaptiveBRIM (50). The modularity is additionally normalized according to a maximum theoretical modularity as detailed in reference 51.

### Choosing life history traits for coexistence.

The life history traits for a given interaction network are chosen to ensure that all microbial host and virus populations can coexist, adapted from reference 52.

First, we sample target fixed-point densities *H*_i_^*^ and *V*_i_^*^ for each microbial host and virus population. In addition, we sample adsorption rates ϕ_*ij*_ and burst sizes β_*ij*_. All of these parameters are independently and randomly sampled from uniform distributions with biologically feasible ranges specified in Table 1. We use a fixed carrying capacity density *K* = 10^6^ cells/ml for all parameter sets.

**TABLE 1:** Sampling ranges for parameters in the virus-microbe dynamic model (equations 1 and 2)

Parameter variable	Parameter	Sampling range	Units
*H*_i_^*^	Host *i* target steady-state density	10^3^–10^4^	No. of cells/milliliter (ml)
*V*_i_^*^	Virus *j* target steady-state density	10^6^–10^7^	No. of virions/ml
*K*	Community-wide host carrying capacity	10^6^	No. of cells/ml
ϕ_*ij*_	Adsorption rate of virus *j* into host *i*	10^−7^–10^−6^	ml/day
β_*ij*_	Burst size of virus *j* per host *i*	10–100	No. of virions/cell
*H*_*i*_^0*^	Host *i* target steady-state density in the absence of viruses	10^3^–10^6^	No. of cells/ml
*a_ii′_*	Competitive effect of host *i*′ on host *i*	0–1	

Next, we sample microbe-microbe competition terms *a*_*ii*′_. We introduce an additional constraint that microbial populations should coexist in the absence of all viruses. To this end, we sample target virus-free fixed-point densities *H*_*i*_^0*^ from a uniform distribution with a range specified in Table 1. After sampling, the *H*_*i*_^0*^ remains fixed. According to equation 1, coexistence in the virus-free setting is satisfied when

(3) $$K=\sum_{i\prime }^{N_{H}}a_{ii\prime }H_{i\prime }^{0*}$$

for each microbial host *i*. To start, we set all intraspecific competition to one (*a_ii_* = 1) and all interspecific competition to zero (*a_ii_*_′_ = 0 for *i*′ ≠ *i*). Then, for each microbial host *i*, we randomly choose an index *k* ≠ *i* and sample *a_ik_* uniformly between zero and one. If the updated sum in equation 3 does not exceed the carrying capacity *K*, we repeat for a new index *k*. Once the carrying capacity is exceeded, we adjust the most recent *a_ik_* so that equation 3 is satisfied exactly.

Finally, the viral decay rates *m*_*j*_ and host growth rates *r*_*i*_ are computed from the fixed-point versions of equations 1 and 2:

(4) $$m_{j}=\sum_{i}^{N_{H}}M_{ij}\phi_{ij}\beta_{ij}H_{i}^{*}$$

(5) $$r_{i}=\left(\sum_{j}^{N_{V}}M_{ij}\phi_{ij}V_{j}^{*}\right)/\left(1-\frac{\sum_{i\prime }^{N_{H}}a_{ii\prime }H_{i\prime }^{*}}{K}\right)$$

### Simulating and sampling time series.

We use Matlab’s native ODE45 function to numerically simulate the virus-microbe dynamic model specified above in “Dynamic model of a virus-microbe community” with interaction network and life history traits generated as described in “Generating interaction networks and characterizing network structure” and “Choosing life history traits for coexistence” above. We use a relative error tolerance of 10^−8^. Initial conditions are chosen by perturbing the fixed-point densities *H*_i_^*^ and *V*_i_^*^ by a multiplicative factor δ where the sign of δ is chosen randomly for each microbial host and virus population. We note that δ can be used to tune the amount of variability in the simulated time series (see Fig. S1 in the supplemental material).

After simulating virus and microbe time series, we sample the time series at regularly spaced sample times (every 2 h) for a fixed duration (200 h, or 100 samples). Therefore, for each virus and each microbe in the community, we take *S* samples at times *t*_1_,…,*t*_*S*_. We use the same sampling frequency and the same *S* for each inference method, except for time-delayed correlation (see “Standard and time-delayed Pearson correlation networks” below).

### Standard and time-delayed Pearson correlation networks.

We assume *S* regularly spaced sample times *t*_1_,…,*t*_*S*_for each host type *H*_*i*_ and each virus type *V*_*j*_. The samples are log transformed, that is $h_{i}(t_{k})=\log_{10}H_{i}(t_{k})$ and $v_{j}(t_{k})=\log_{10}V_{j}(t_{k})$ for each sampled time point *t*_*k*_. The standard Pearson correlation coefficient between host *i* and virus *j* is then

(6) $$r_{ij}=\frac{\overset{S}{\underset{k=\;1}{\sum }}\left[h_{i}\left(t_{k}^{}\right)-{\overset{¯}{h}}_{i}\right]\left[v_{j}\left(t_{k}\right)-{\overset{¯}{v}}_{j}\right]}{\sqrt{\overset{S}{\underset{k=\;1}{\sum }}{\left[h_{i}\left(t_{k}\right)-{\overset{¯}{h}}_{i}\right]}^{2}}\sqrt{\overset{S}{\underset{k=\;1}{\sum }}{\left[v_{j}\left(t_{k}\right)-{\overset{¯}{v}}_{j}\right]}^{2}}}$$

where ${\overset{¯}{h}}_{i}=\frac{1}{S}\overset{S}{\underset{k=\;1}{\sum }}h_{i}\left(t_{k}\right)$ and ${\overset{¯}{v}}_{j}=\frac{1}{S}\overset{S}{\underset{k=\;1}{\sum }}v_{j}\left(t_{k}\right)$ are the sample means. The correlation coefficients for all virus-host pairs are represented as a bipartite matrix **R** of size *N_H_* × *N_V_* analogous to the interaction network (see “Generating interaction networks and characterizing network structure” above).

Time-delayed correlations are computed by sampling the virus time series later in time. Each virus-host pair may have a unique time delay τ_*ij*_. For example, if host *i* is sampled at times *t*_1_,…,*t*_*S*_, then virus *j* is sampled at times *t*_1_ + τ_*ij*_,…,*t*_*S*_ + τ_*ij*_. We keep the number of samples *S* fixed, and consequently allow virus *j* to be sampled beyond the final sample time *t*_*S*_ of the hosts. The time-delayed Pearson correlation coefficient is

(7) $$r_{ij}^{\tau }=\frac{\overset{S}{\underset{k=\;1}{\sum }}\left[h_{i}\left(t_{k}\right)-{\overset{¯}{h}}_{i}\right]\left[v_{j}(t_{k}+\tau_{ij})-{\overset{¯}{v}}_{j}^{\tau_{ij}}\right]}{\sqrt{\overset{S}{\underset{k=\;1}{\sum }}{\left[h_{i}\left(t_{k}\right)-{\overset{¯}{h}}_{i}\right]}^{2}}\sqrt{\overset{S}{\underset{k=1}{\sum }}{\left[v_{j}(t_{k}+\tau_{ij})-{\overset{¯}{v}}_{j}^{\tau_{ij}}\right]}^{2}}}$$

where ${\bar{v}}_{j}^{\tau_{ij}}=\frac{1}{S}\sum_{k=\;1}^{S}v_{j}(t_{k}+\tau_{ij})$ is the mean of the time-delayed virus sample. As before, the correlation coefficients for all virus-host pairs is a bipartite matrix **R**^τ^ of size *N_H_* × *N*_*V*_.

Pearson correlation coefficients, as specified above, were computed using Matlab’s native Corr function with type pearson. Alternate correlation types, including Spearman correlation and Kendall correlation, are also supported by the Corr function and are utilized in the supplemental material.

### eLSA networks.

Extended local similarity analysis (eLSA) is a correlation-based inference method that is widely used with *in situ* time series of complex microbial communities (32–38). eLSA attempts to detect local correlations, that is, time series that trend together for only a portion of the sample period. In addition, eLSA allows for time-delayed correlations (as described in the previous section “Standard and time-delayed Pearson correlation networks”). To this end, a local similarity (LS) score is computed for each pair of time series. The LS score is analogous to computing the Pearson correlation for all possible subsections of the two time series, with offsets up to a predecided length, and keeping the maximum absolute correlation. As an example, two time series may trend strongly during the first half of the sample period but not during the second half. For such a pair of time series, the Pearson correlation would be low, but the LS score would be high.

To compute the LS score, the two time series are first transformed to have normal distributions (we note that such a transformation is nonstationary and thus may induce spurious correlations). The LS score is the maximal sum of the product of the entries across all possible subsections, normalized by the time series length. If a predefined delay is specified, the subsections are additionally offset from one another from zero up to the delay amount (29–31).

We applied eLSA to our simulated time series data. We used samples of all *N*_*H*_ host types and all *N*_*V*_ virus types with *S* regularly spaced sample times *t*_1_,…,*t*_*S*_as input. We used the lsa-compute.py Python script and set parameters to specify the number of sampled points (spotNum = S), number of replicates (repNum = 1), number of bootstraps (b = 0), and number of permutations (x = 1). All other parameters were left with their default settings, including the maximum allowed time delay (delayLimit = 3). The lsa-compute.py script computes eLSA scores between all virus-host, host-host, and virus-virus pairs. We selected only the virus-host eLSA scores and arranged them in a bipartite matrix of size *N*_*H*_ × *N*_*V*_ analogous to the interaction network (see “Generating interaction networks and characterizing network structure” above). We used a custom Matlab script write_elsa.m to generate “.csv” data files in the format specified by the eLSA documentation. We used a custom bash script elsa_compute_all.sh to run the eLSA analysis on the ensemble of virus-microbe communities. Finally, we used a custom Matlab script read_elsa.m to import the results into Matlab for scoring (see “Scoring correlation network accuracy” below).

### SparCC networks.

Sparse correlations for compositional data (SparCC) is a correlation-based inference method for use with compositional time series data. This is relevant for “-omics” data in which abundances are typically relative. It is well-known that compositional data pose challenges for standard statistics, including Pearson correlation and other types of correlation. Because the data sum to one, individual time series are not independent. This biases correlations to be negative regardless of the trend between the underlying absolute abundances. SparCC estimates the Pearson correlation between two time series while taking into account these compositional dependencies. In particular, SparCC computes the variance of the log-transformed ratio of two time series and compares this quantity to the variances of the individual log-transformed time series. SparCC assumes sparsity in the correlation matrix but is robust to violations of this assumption (40).

We applied SparCC to our simulated time series data as a means to evaluate correlation-based inference in a scenario in which underlying viral and microbial densities can be measured only relatively. Given samples at *S* regularly spaced sample times *t*_1_,…,*t*_*S*_, we first normalized the *N*_*H*_ host types and *N*_*V*_ virus types at each sample time *t*_*k*_ by

(8) $$N_{H,k}=\overset{N_{H}}{\underset{i=\;1}{\sum }}H_{i}\left(t_{k}\right)$$

for the hosts and by

(9) $$N_{V,k}=\overset{N_{V}}{\underset{j=\;1}{\sum }}V_{j}\left(t_{k}\right)$$

for the viruses. We used the normalized *N*_*H*_ host and *N*_*V*_ virus samples as input for the SparCC computation using the SparCC.py script. All parameters were left with their default settings. We used a custom Matlab script write_sparcc.m to generate “.csv” data files in the format specified by the SparCC documentation. We used a custom bash script sparcc_compute_all.sh to run the SparCC analysis on the ensemble of virus-microbe communities. Finally, we used a custom Matlab script read_sparcc.m to import the results into Matlab for scoring (see “Scoring correlation network accuracy”).

### Scoring correlation network accuracy.

To evaluate how well the Pearson correlation, eLSA, or SparCC (collectively referred to as “correlation”) network **R** recapitulates the original interaction network **M̃**, we compute the receiver operator curve (ROC). First, we binarize the interaction network **M̃** so that it is a Boolean network **M** of zeros (noninteractions) and ones (interactions). Then we choose a threshold of interaction *c* between the minimum and maximum attainable values of the correlation network **R**; for Pearson correlation, these values are −1 and +1. Correlations in **R** that are greater than or equal to *c* are categorized as interactions (ones), while those that are less are noninteractions (zeros). The true-positive (TP) count is the number of interactions in **M** correctly predicted by the thresholded correlation network **R**_c_. The false-positive (FP) count is the number of noninteractions in **M** incorrectly predicted by **R**_c_. The TP and FP counts are normalized by the number of interactions and noninteractions in **M** to obtain the true-positive rate (TPR) and false-positive rate (FPR). TPR and FPR are computed for all thresholds *c* to obtain the receiver operator curve (ROC).

The overall “score” of the correlation network **R** is the area under the curve (AUC). A perfect prediction results in AUC = 1, since for some threshold, TPR = 1 and FPR = 0. Random predictions result in AUC = 1/2, since TPR = FPR across all possible thresholds. AUC values which are less than 1/2 indicate a misclassification of “interaction,” that is, categorizing interactions and noninteractions in the opposite way would have resulted in a better prediction of **M̃**.

### Availability of data and materials.

Analysis was primarily performed in Matlab. All Matlab scripts, Matlab data files (also available as “.csv” files), and custom bash scripts for implementing eLSA and SparCC are publicly available on GitHub (https://github.com/WeitzGroup/correlation_based_inference) and archived on Zenodo (DOI 10.5281/zenodo.844918). The BiMat Matlab package (48) used for characterizing bipartite networks is available on GitHub (https://github.com/cesar7f/BiMat). The eLSA Python package (29–31) is available on Bitbucket (https://bitbucket.org/charade/elsa/wiki/Home). The SparCC Python package (40) is available on Bitbucket (https://bitbucket.org/yonatanf/sparcc).
