# Supplementary figures and images for: Limitations of Correlation-Based Inference in Complex Virus-Microbe Communities
Source: mSystems. 2018 Aug 28;3(4):e00084-18. doi: 10.1128/mSystems.00084-18 (PMC6113591; doi:10.1128/mSystems.00084-18)

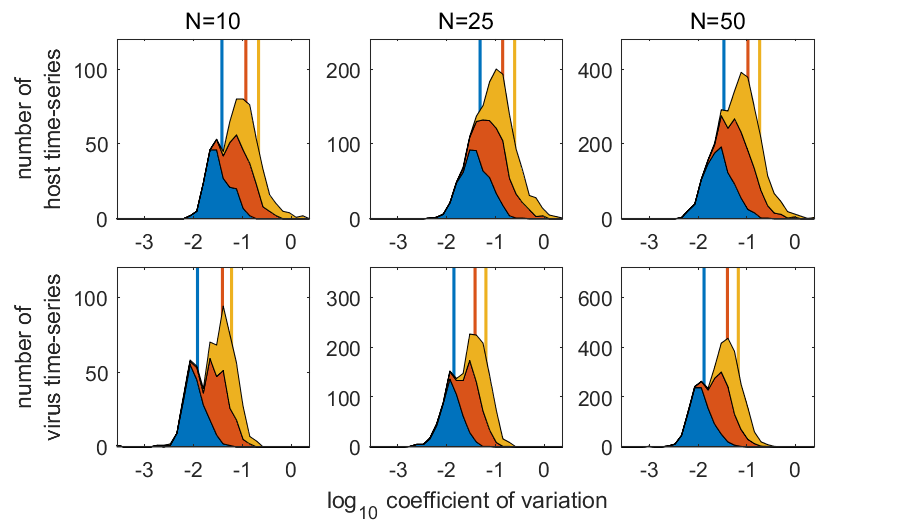

Supplement: FIG S1 [file sys004182254sf1.tif]

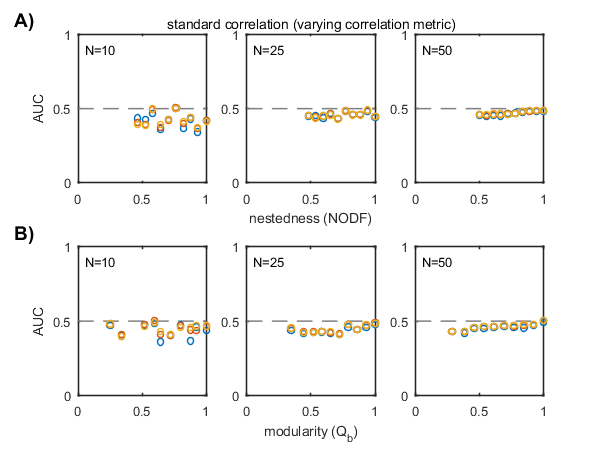

Supplement: FIG S2 [file sys004182254sf2.tif]

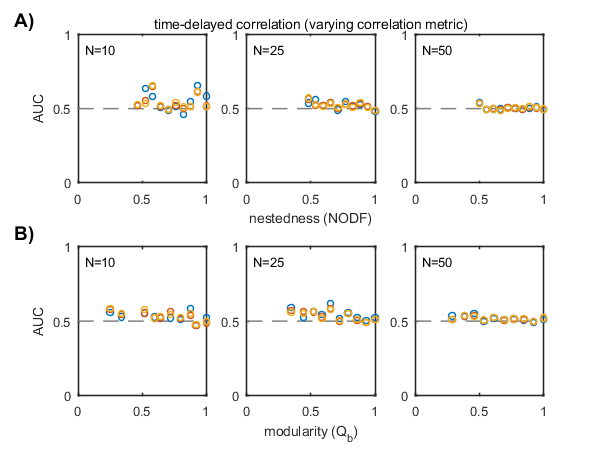

Supplement: FIG S3 [file sys004182254sf3.tif]

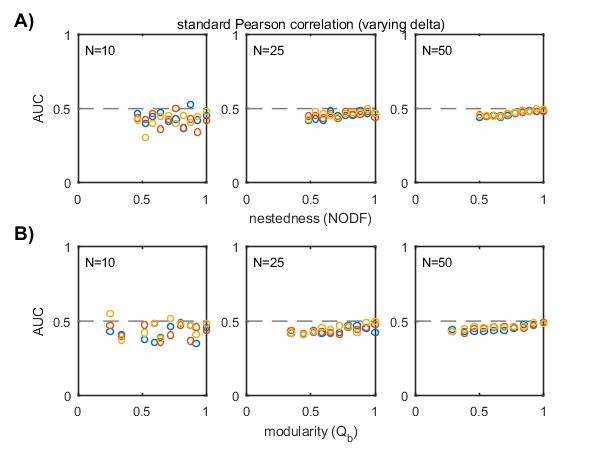

Supplement: FIG S4 [file sys004182254sf4.tif]

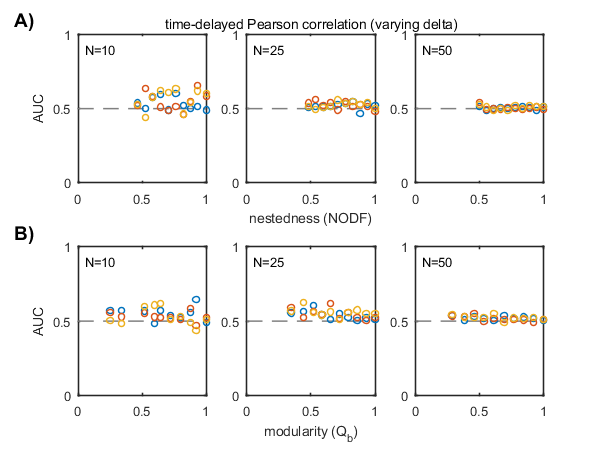

Supplement: FIG S5 [file sys004182254sf5.tif]

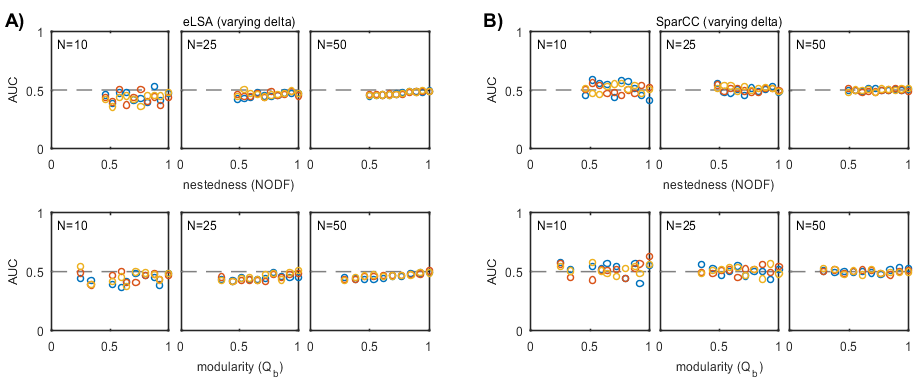

Supplement: FIG S6 [file sys004182254sf6.tif]

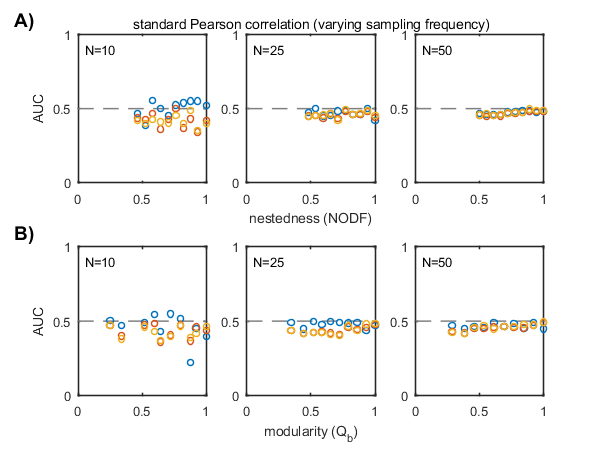

Supplement: FIG S7 [file sys004182254sf7.tif]

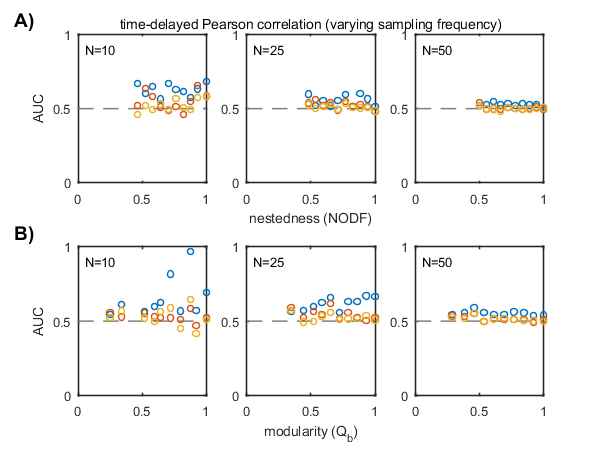

Supplement: FIG S8 [file sys004182254sf8.tif]

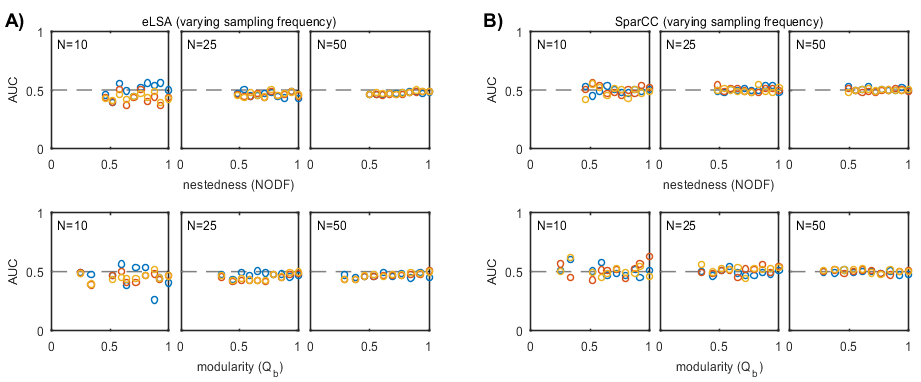

Supplement: FIG S9 [file sys004182254sf9.tif]
